# Structure-Function Analysis of Diacylglycerol Acyltransferase Sequences from 70 Organisms

**DOI:** 10.1186/1756-0500-4-249

**Published:** 2011-07-21

**Authors:** Heping Cao

**Affiliations:** 1 Commodity Utilization Research Unit, Southern Regional Research Center, Agricultural Research Service, U.S. Department of Agriculture, 1100 Robert E. Lee Blvd., New Orleans, Louisiana 70124, USA

## Abstract

**Background:**

Diacylglycerol acyltransferase families (DGATs) catalyze the final and rate-limiting step of triacylglycerol (TAG) biosynthesis in eukaryotic organisms. Understanding the roles of DGATs will help to create transgenic plants with value-added properties and provide clues for therapeutic intervention for obesity and related diseases. The objective of this analysis was to identify conserved sequence motifs and amino acid residues for better understanding of the structure-function relationship of these important enzymes.

**Results:**

117 DGAT sequences from 70 organisms including plants, animals, fungi and human are obtained from database search using tung tree DGATs. Phylogenetic analysis separates these proteins into DGAT1 and DGAT2 subfamilies. These DGATs are integral membrane proteins with more than 40% of the total amino acid residues being hydrophobic. They have similar properties and amino acid composition except that DGAT1s are approximately 20 kDa larger than DGAT2s. DGAT1s and DGAT2s have 41 and 16 completely conserved amino acid residues, respectively, although only two of them are shared by all DGATs. These residues are distributed in 7 and 6 sequence blocks for DGAT1s and DGAT2s, respectively, and located at the carboxyl termini, suggesting the location of the catalytic domains. These conserved sequence blocks do not contain the putative neutral lipid-binding domain, mitochondrial targeting signal, or ER retrieval motif. The importance of conserved residues has been demonstrated by site-directed and natural mutants.

**Conclusions:**

This study has identified conserved sequence motifs and amino acid residues in all 117 DGATs and the two subfamilies. None of the completely conserved residues in DGAT1s and DGAT2s is present in recently reported isoforms in the multiple sequences alignment, raising an important question how proteins with completely different amino acid sequences could perform the same biochemical reaction. The sequence analysis should facilitate studying the structure-function relationship of DGATs with the ultimate goal to identify critical amino acid residues for engineering superb enzymes in metabolic engineering and selecting enzyme inhibitors in therapeutic application for obesity and related diseases.

## Background

The complete genomes of many organisms including human, mouse, *Arabidopsis *and rice have been sequenced. The immediate challenge of post-genomic biology is to determine the biological functions of proteins encoded by unknown genes. Many endogenous proteins occur in extremely low abundance (such as the anti-inflammatory protein tristetraprolin/zinc-finger protein 36, TTP/ZFP36) [1] and are labile (such as omega-3 fatty-acid desaturase, FAD3) [2], which complicates characterization of those proteins.

One approach to gain clues about the structure-function relationship of proteins is to perform comprehensive amino acid sequence analysis. It is generally accepted that critical amino acid residues and sequence motifs in the same family of proteins are evolutionarily conserved. We previously used a protein sequence analysis approach to identify conserved sequence motifs and critical amino acid residues in several families of proteins from diverse organisms. The protein sequences we analyzed previously include the TTP/ZFP36 family involved in mRNA binding and destabilization [3,4], adenylate translocators [5], starch/glycogen synthases [6], starch/glycogen branching enzymes [7,8], and starch/glycogen debranching enzymes [9].

Triacylglycerols (TAGs) are the major molecules of energy storage in eukaryotes. They also serve as a reservoir of fatty acids for membrane biogenesis and lead to obesity due to excessive accumulation in adipose tissues. Diacylglycerol acyltransferase families (DGATs) are integral microsomal membrane proteins that catalyze the last and rate-limiting step of TAG biosynthesis in eukaryotic organisms. DGATs esterify *sn*-1,2-diacylglycerol with a long-chain fatty acyl-CoA. DGAT genes have been isolated from many organisms. At least two forms of DGATs are present in mammals [10,11] and plants [12,13] with additional forms reported in burning bush (*Euonymus alatus) *[14], peanut [15] and *Arabidopsis *[16]. Plants and animals deficient in DGATs accumulate less TAG [17-19]. Animals with reduced DGAT activity are resistant to diet-induced obesity [18,20] and lack milk production [18]. Over-expression of DGAT enzymes increases TAG content in plants [14,21-26], animals [27-30] and yeast [31]. DGATs have nonredundant functions in TAG biosynthesis in species such as mice [19] and tung tree (*Vernicia fordii*) [13]. Mice deficient in DGAT1 are viable, have modest decreases in TAG, and are resistant to diet-induced obesity [18,32]. In contrast, mice deficient in DGAT2 have severe reduction of TAG and die shortly after birth [19]. The fact that DGAT1 is unable to compensate for the deficiency in DGAT2 knockout mice indicates the nonredundant functions of each DGAT isoform in TAG biosynthesis during mammal development. Therefore, understanding the roles of DGATs in plants and animals will have tremendous potential in creating new oilseed crops with value-added properties and providing information for therapeutic intervention for obesity and related diseases.

Limited numbers of DGAT amino acid sequences were analyzed previously [13,33]. However, there is a lack of comprehensive analysis of amino acid sequences of DGATs among diverse organisms. The objective of this analysis was to identify conserved sequence motifs and amino acid residues in 117 DGATs from 70 organisms to provide a better understanding of the structure-function relationship of these important enzymes.

## Results

### Phylogenetic analysis and classification of DGATs

**Figure 1:**
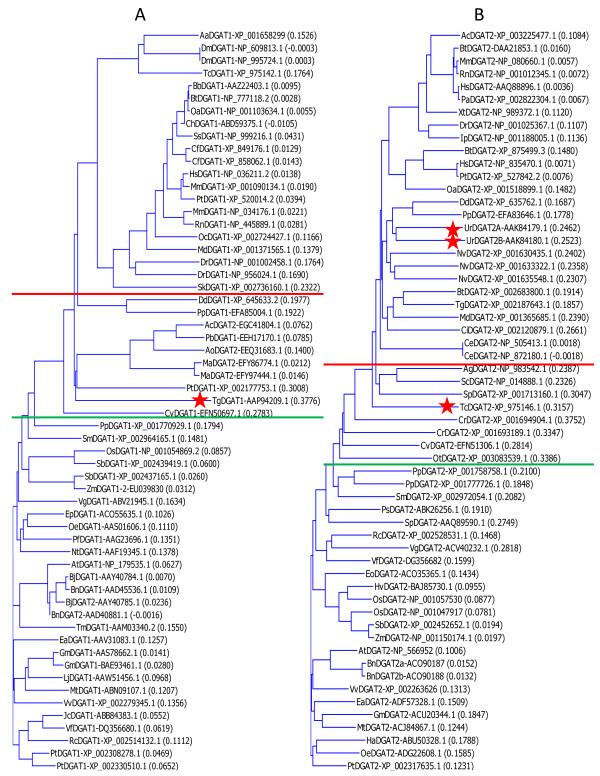
**Phylogenetic analysis of DGAT1s and DGAT2s**. The presumed evolutionary relationships among the 117 DGATs from 70 organisms (listed in Table 1) were analyzed by phylogenetic analysis based on the Neighbor-Joining method of Saitou and Nei. The numbers in the parenthesis following DGAT names are the calculated distance values which reflect the degree of divergence between all pairs of DGAT sequences analyzed. The sequences above the red line are from animals, whereas the sequences below the green line are from plants. A red "star" before the sequence indicates the exceptional sequence from the grouping.

A database search using tung tree (*Vernicia fordii*) DGAT1 and DGAT2 protein sequences [13] has identified 117 DGAT sequences from 70 organisms including plants, animals, fungi and human (Table 1). More than two forms of DGATs are present in a number of species. For example, *Bos taurus *(cow) and *Brassica napus *(rape) have four forms of DGATs, whereas *Homo sapiens *(human) and *Danio rerio *(zebrafish) have three forms of DGATs (Table 1). Phylogenetic analysis indicates that all 117 DGAT protein sequences are grouped in the same phylogenetic tree (data not shown) and clearly separated into DGAT1 and DGAT2 subfamilies (Figure 1). DGAT1s are more conserved than DGAT2s. DGATs from plants, animals and fungi are also distinctly separated from each other with a few exceptions (Figure 1).

**Table 1:** DGATs sequence information.

**No**.	DGAT name	Organism	DGAT	GenBank #	**No**.	DGAT name	Organism	DGAT	GenBank #
**1**	AaDGAT1-XP_001658299 *	*Aedes aegypti *(A) ***	1	XP_001658299	**60**	NtDGAT1-AAF19345.1	*Nicotiana tabacum *(P, tobacco)	1	AAF19345.1
**2**	AcDGAT1-EGC41804.1 **	*Ajellomyces capsulatus *(F)	1	EGC41804.1	**61**	NvDGAT2a-XP_001630435.1	*Nematostella vectensis *(A, worm)	2a	XP_001630435.1
**3**	AcDGAT2-XP_003225477.1	*Anolis carolinensis *(A)	2	XP_003225477.1	**62**	NvDGAT2b-XP_001633322.1	*Nematostella vectensis *(A, worm)	2b	XP_001633322.1
**4**	AgDGAT2-NP_983542.1	*Ashbya gossypii *(F)	2	NP_983542.1	**63**	NvDGAT2c-XP_001635548.1	*Nematostella vectensis *(A, worm)	2c	XP_001635548.1
**5**	AoDGAT1-EEQ31683.1 **	*Arthroderma otae *(F)	1	EEQ31683.1	**64**	OaDGAT1-NP_001103634.1	*Ovis aries *(A, sheep)	1	NP_001103634.1
**6**	AtDGAT1-NP_179535.1	*Arabidopsis thaliana *(P)	1	NP_179535.1	**65**	OaDGAT2-XP_001518899.1 *	*Ovis aries *(A, sheep)	2	XP_001518899.1
**7**	AtDGAT2-NP_566952	*Arabidopsis thaliana *(P)	2	NP_566952	**66**	OcDGAT1-XP_002724427.1	*Oryctolagus cuniculus *(A, rabbit)	1	XP_002724427.1
**8**	BbDGAT1-AAZ22403.1	*Bubalus bubalis *(A, buffalo)	1	AAZ22403.1	**67**	OeDGAT1-AAS01606.1	*Olea europaea *(P, tree)	1	AAS01606.1
**9**	BjDGAT1a-AAY40784.1	*Brassica juncea *(P)	1a	AAY40784.1	**68**	OeDGAT2-ADG22608.1	*Olea europaea *(P, tree)	2	ADG22608.1
**10**	BjDGAT1b-AAY40785.1 **	*Brassica juncea *(P)	1b	AAY40785.1	**69**	OsDGAT1-NP_001054869.2	*Oryza sativa *(P, rice)	1	NP_001054869.2
**11**	BnDGAT1a-AAD45536.1	*Brassica napus *(P)	1a	AAD45536.1	**70**	OsDGAT2a-NP_001047917	*Oryza sativa *(P, rice)	2a	NP_001047917
**12**	BnDGAT1b-AAD40881.1 *, **	*Brassica napus *(P)	1b	AAD40881.1	**71**	OsDGAT2b-NP_001057530	*Oryza sativa *(P, rice)	2b	NP_001057530
**13**	BnDGAT2a-ACO90187	*Brassica napus *(P)	2	ACO90187	**72**	OtDGAT2-XP_003083539.1	*Ostreococcus tauri *(Algae)	2	XP_003083539.1
**14**	BnDGAT2b-ACO90188	*Brassica napus *(P)	2	ACO90188	**73**	PaDGAT2-XP_002822304.1	*Pongo abelii *(A, Sumatran orangutan)	2	XP_002822304.1
**15**	BtDGAT1-NP_777118.2	*Bos taurus *(A, cattle)	1	NP_777118.2	**74**	PbDGAT1-EEH17170.1	*Paracoccidioides brasiliensis *(F)	1	EEH17170.1
**16**	BtDGAT2a-DAA21853.1	*Bos taurus *(A, cattle)	2a	DAA21853.1	**75**	PfDGAT1-AAG23696.1	*Perilla frutescens *(P)	1	AAG23696.1
**17**	BtDGAT2b-XP_875499.3	*Bos taurus *(A, cattle)	2b	XP_875499.3	**76**	PpDGAT1-EFA85004.1	*Polysphondylium pallidum *(F, mold)	1	EFA85004.1
**18**	BtDGAT2c-XP_002683800.1 *	*Bos taurus *(A, cattle)	2c	XP_002683800.1	**77**	PpDGAT2-EFA83646.1	*Polysphondylium pallidum *(F, mold)	2	EFA83646.1
**19**	CeDGAT2a-NP_505413.1	*Caenorhabditis elegans *(A, worm)	2a	NP_505413.1	**78**	PpDGAT1-XP_001770929.1	*Physcomitrella patens *(P, moss)	1	XP_001770929.1
**20**	CeDGAT2b-NP_872180.1	*Caenorhabditis elegans *(A, worm)	2b	NP_872180.1	**79**	PpDGAT2a-XP_001758758.1	*Physcomitrella patens *(P, moss)	1	XP_001758758.1
**21**	CfDGAT1b-XP_849176.1	*Canis familiaris *(A, dog)	1b	XP_849176.1	**80**	PpDGAT2b-XP_001777726.1	*Physcomitrella patens *(P, moss)	2b	XP_001777726.1
**22**	CfDGAT1c-XP_858062.1	*Canis familiaris *(A, dog)	1c	XP_858062.1	**81**	PsDGAT2-ABK26256.1	*Picea sitchensis *(P, tree)	2	ABK26256.1
**23**	ChDGAT1-ABD59375.1 *	*Capra hircus *(A, sheep)	1	ABD59375.1	**82**	PtDGAT1-XP_520014.2	*Pan troglodytes *(A, chimpanzee)	1	XP_520014.2
**24**	CiDGAT2-XP_002120879.1	*Ciona intestinalis *(A)	2	XP_002120879.1	**83**	PtDGAT2-XP_527842.2	*Pan troglodytes *(A, chimpanzee)	2	XP_527842.2
**25**	CrDGAT2a-XP_001694904.1	*Chlamydomonas reinhardtii *(Algae)	2a	XP_001694904.1	**84**	PtDGAT1-XP_002177753.1 *	*Phaeodactylum tricornutum *(F)	1	XP_002177753.1
**26**	CrDGAT2b-XP_001693189.1	*Chlamydomonas reinhardtii *(Algae)	2b	XP_001693189.1	**85**	PtDGAT1a-XP_002308278.1	*Populus trichocarpa *(P, tree)	1a	XP_002308278.1
**27**	CvDGAT1- EFN50697.1	*Chlorella variabilis *(Algae)	1	EFN50697.1	**86**	PtDGAT1b-XP_002330510.1	*Populus trichocarpa *(P, tree)	1b	XP_002330510.1
**28**	CvDGAT2-EFN51306.1 *	*Chlorella variabilis *(Algae)	2	EFN51306.1	**87**	PtDGAT2-XP_002317635.1 *	*Populus trichocarpa *(P, tree)	2	XP_002317635.1
**29**	DdDGAT1- XP_645633.2	*Dictyostelium discoideum *(mold)	1	XP_645633.2	**88**	RcDGAT1-XP_002514132.1	*Ricinus communis *(P, castor bean)	1	XP_002514132.1
**30**	DdDGAT2-XP_635762.1	*Dictyostelium discoideum *(mold)	2	XP_635762.1	**89**	RcDGAT2-XP_002528531.1	*Ricinus communis *(P, castor bean)	1	XP_002528531.1
**31**	DmDGAT1a-NP_609813.1	*Drosophila melanogaster *(A, fly)	1a	NP_609813.1	**90**	RnDGAT1-NP_445889.1	*Rattus norvegicus *(A, rat)	1	NP_445889.1
**32**	DmDGAT1d-NP_995724.1	*Drosophila melanogaster *(A, fly)	1d	NP_995724.1	**91**	RnDGAT2-NP_001012345.1	*Rattus norvegicus *(A, rat)	2	NP_001012345.1
**33**	DrDGAT1a-NP_956024.1	*Danio rerio *(A, zebrafish)	1a	NP_956024.1	**92**	SbDGAT1a-XP_002437165.1	*Sorghum bicolor *(P, sorghum)	1a	XP_002437165.1
**34**	DrDGAT1b-NP_001002458.1	*Danio rerio *(A, zebrafish)	1b	NP_001002458.1	**93**	SbDGAT1b-XP_002439419.1	*Sorghum bicolor *(P, sorghum)	1b	XP_002439419.1
**35**	DrDGAT2-NP_001025367.1	*Danio rerio *(A, zebrafish)	2	NP_001025367.1	**94**	SbDGAT2-XP_002452652.1	*Sorghum bicolor *(P, sorghum)	2	XP_002452652.1
**36**	EaDGAT1-AAV31083.1	*Euonymus alatus *(P)	1	AAV31083.1	**95**	ScDGAT2-NP_014888.1	*Saccharomyces cerevisiae *(F, yeast)	2	NP_014888.1
**37**	EaDGAT2-ADF57328.1	*Euonymus alatus *(P)	2	ADF57328.1	**96**	SkDGAT1-XP_002736160.1	*Saccoglossus kowalevskii *(A, worm)	1	XP_002736160.1
**38**	EoDGAT2-ACO35365.1	*Elaeis oleifera *(P)	2	ACO35365.1	**97**	SmDGAT1-XP_002964165.1	*Selaginella moellendorffii *(P)	1	XP_002964165.1
**39**	EpDGAT1-ACO55635.1	*Echium pitardii *(P)	1	ACO55635.1	**98**	SmDGAT2-XP_002972054.1	*Selaginella moellendorffii *(P)	2	XP_002972054.1
**40**	GmDGAT1a-AAS78662.1	*Glycine max *(P, soybean)	1a	AAS78662.1	**99**	SpDGAT2-AAQ89590.1	*Spirodela polyrhiza *(P)	2	AAQ89590.1
**41**	GmDGAT1b-BAE93461.1	*Glycine max *(P, soybean)	1b	BAE93461.1	**100**	SpDGAT2-XP_001713160.1	*Schizosaccharomyces pombe *(F, yeast)	2	XP_001713160.1
**42**	GmDGAT2-ACU20344.1	*Glycine max *(P, soybean)	2	ACU20344.1	**101**	SsDGAT1-NP_999216.1	*Sus scrofa *(A, pig)	1	NP_999216.1
**43**	HaDGAT2-ABU50328.1	*Helianthus annuus *(P)	2	ABU50328.1	**102**	TcDGAT1-XP_975142.1	*Tribolium castaneum *(A)	1	XP_975142.1
**44**	HsDGAT1-NP_036211.2	*Homo sapiens *(human)	1	NP_036211.2	**103**	TcDGAT2-XP_975146.1	*Tribolium castaneum *(A)	2	XP_975146.1
**45**	HsDGAT2a-AAQ88896.1	*Homo sapiens *(human)	2a	AAQ88896.1	**104**	TgDGAT1-AAP94209.1	*Toxoplasma gondii *(A, worm)	1	AAP94209.1
**46**	HsDGAT2b-NP_835470.1	*Homo sapiens *(human)	2b	NP_835470.1	**105**	TgDGAT2-XP_002187643.1	*Taeniopygia guttata *(A, bird)	2	XP_002187643.1
**47**	HvDGAT2-BAJ85730.1	*Hordeum vulgare *(P, barley)	2	BAJ85730.1	**106**	TmDGAT1-AAM03340.2	*Tropaeolum majus *(P)	1	AAM03340.2
**48**	IpDGAT2b-NP_001188005.1	*Ictalurus punctatus *(A, catfish)	2b	NP_001188005.1	**107**	UrDGAT2A-AAK84179.1	*Umbelopsis ramanniana *(F)	2a	AAK84179.1
**49**	JcDGAT1-ABB84383.1	*Jatropha curcas *(P)	1	ABB84383.1	**108**	UrDGAT2B-AAK84180.1	*Umbelopsis ramanniana *(F)	2b	AAK84180.1
**50**	LjDGAT1-AAW51456.1	*Lotus japonicas *(P)	1	AAW51456.1	**109**	VfDGAT1-DQ356680.1	*Vernicia fordii *(P, tung tree)	1	DQ356680.1
**51**	MaDGAT1a-EFY86774.1 **	*Metarhizium acridum *(F)	1a	EFY86774.1	**110**	VfDGAT2-DQ356682.1	*Vernicia fordii *(P, tung tree)	2	DQ356682
**52**	MaDGAT1b-EFY97444.1 **	*Metarhizium anisopliae *(F)	1b	EFY97444.1	**111**	VgDGAT1-ABV21945.1	*Vernonia galamensis *(P)	1	ABV21945.1
**53**	MdDGAT1-XP_001371565.1	*Monodelphis domestica *(A, opossum)	1	XP_001371565.1	**112**	VgDGAT2-ACV40232.1	*Vernonia galamensis *(P)	2	ACV40232.1
**54**	MdDGAT2-XP_001365685.1	*Monodelphis domestica *(A, opossum)	2	XP_001365685.1	**113**	VvDGAT1-XP_002279345.1	*Vitis vinifera *(P, grape)	1	XP_002279345.1
**55**	MmDGAT1-NP_034176.1	*Mus musculus *(A, mouse)	1	NP_034176.1	**114**	VvDGAT2-XP_002263626	*Vitis vinifera *(P, grape)	2	XP_002263626
**56**	MmDGAT2-NP_080660.1	*Mus musculus *(A, mouse)	2	NP_080660.1	**115**	XtDGAT2-NP_989372.1	*Xenopus tropicalis *(A, frog)	2	NP_989372.1
**57**	MmDGAT1-XP_001090134.1	*Macaca mulatta *(A, monkey)	1	XP_001090134.1	**116**	ZmDGAT1b-EU039830	*Zea mays *(P, corn)	1b	EU039830
**58**	MtDGAT1-ABN09107.1	*Medicago truncatula *(P)	1	ABN09107.1	**117**	ZmDGAT2-NP_001150174.1	*Zea mays *(P, corn)	2	NP_001150174.1
**59**	MtDGAT2- ACJ84867.1	*Medicago truncatula *(P)	2	ACJ84867.1					

*8 partial sequences were not used for statistics. **6 DGATs named as DGAT2s in the GenBank Databases should be DGAT1s as indicated in the table because they are more similar to DGAT1s. ***A: animal, F: fungus, P: plant.

Six DGATs previously classified as DGAT2s in the GenBank databases [GenBank:EGC41804.1, GenBank:EEQ31683.1, GenBank:AAY40785.1, GenBank:AAD40881.1, GenBank:EFY86774.1 and GenBank:EFY97444.1] are clustered within the DGAT1 subfamily in this phylogenetic analysis (Figure 1). More detailed sequence analysis within the multiple sequence alignment also indicates that their sequences are more similar to DGAT1s (see below). Therefore, these six DGAT2s are reclassified as DGAT1s (AcDGAT1-EGC41804.1, AoDGAT1-EEQ31683.1, BjDGAT1b-AAY40785.1, BnDGAT1b-AAD40881.1, MaDGAT1a-EFY86774.1, and MaDGAT1b-EFY97444.1, respectively) (Table 1).

### DGAT properties and amino acid composition

It is generally observed that DGAT1s are much bigger proteins with more transmembrane domains than DGAT2s. The properties and amino acid composition of 109 full-length DGATs were analyzed and calculated according to the classifications of DGAT1 and DGAT2 subfamilies by phylogenetic analysis. The eight partial sequences listed in Table 1 were excluded from this analysis (AaDGAT1-XP_001658299, BnDGAT1b-AAD40881.1, BtDGAT2c-XP_002683800.1, ChDGAT1-ABD59375.1, CvDGAT2-EFN51306.1, OaDGAT2-XP_001518899.1, PtDGAT1-XP_002177753.1 and PtDGAT2-XP_002317635.1).

DGAT1s have an average of 515 amino acid residues with a standard deviation of 44 amino acid residues among the 55 sequences (Table 2). The average residue number of DGAT1 is 171 amino acids greater than the average DGAT2 length, which has an average of 344 residues with a standard deviation of 29 residues among the 54 sequences (Table 2). This corresponds to approximately 20 kDa difference in the molecular mass. The isoelectric points of DGAT1s and DGAT2s are similar with an average value of 9.17 and 9.28, respectively, however DGAT1s have approximately 4 more charges at pH 7 than DGAT2s (Table 2).

**Table 2:** DGATs properties and amino acid composition (% by frequency)

	DGAT1 (n = 55)	DGAT2 (n = 54)
**Length (aa) **	515 ± 44	344 ± 29
**Molecular weight (Da)**	58796 ± 4871	38920 ± 3330
**Isoelectric point (PI) **	9.17 ± 0.46	9.28 ± 0.42
**Charge at pH 7 **	15.11 ± 5.98	11.34 ± 4.25
**Charged (RKHYCDE) (%) **	26.03 ± 1.27	26.35 ± 2.02
**Acidic (DE) (%) **	7.20 ± 0.90	7.38 ± 0.87
**Basic (KR) (%) **	9.97 ± 0.78	10.47 ± 1.15
**Polar (NCQSTY) (%) **	24.59 ± 2.49	22.25 ± 2.93
**Hydrophobic (AILFWV) (%) **	42.18 ± 2.25	40.92 ± 2.70

The frequency of functional amino acid residue groups between DGAT1 and DGAT2 subfamilies is remarkably similar (Table 2). DGAT1s and DGAT2s have charged residues (RKHYCDE) of approximately 26%, which includes approximately 7% of acidic residues (DE) and 10% of basic residues (KR). The frequency of polar resides (NCQSTY) is also similar with an average of 25% vs. 22% in polar residues for DGAT1s and DGAT2s, respectively. DGAT1s and DGAT2s are integral membrane proteins with 42% and 41% of the total residues being hydrophobic (AILFWV), respectively (Table 2).

### Identification of amino acid residues and sequence motifs conserved among all DGATs

**Figure 2:**
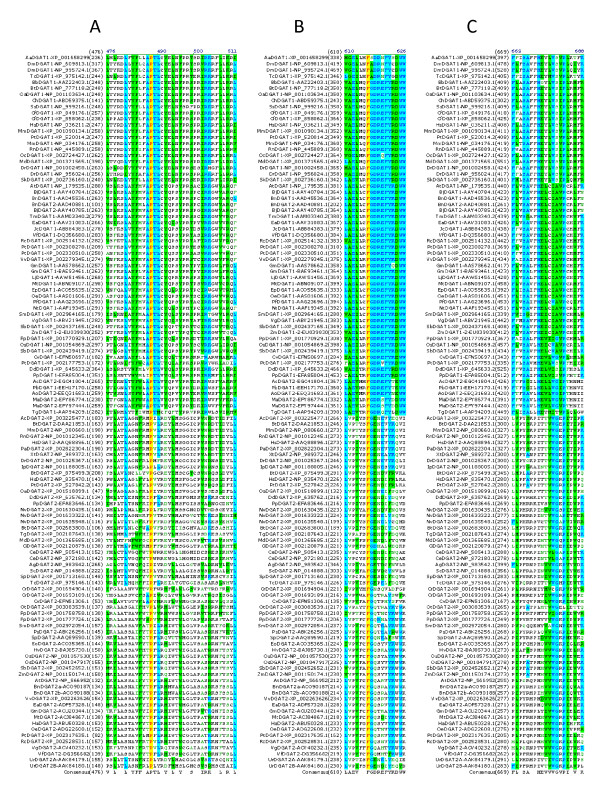
**Conservation of proline and phenylalanine residues in all DGATs**. Multiple sequence alignment was performed using the ClustalW algorithm and 117 DGAT protein sequences from 70 organisms (listed in Table 1). DGAT sequence name is on the left of alignment followed by the start of the amino acid residue of each DGAT protein sequence. The completely conserved proline and phenylalanine residues are highlighted in red on yellow. Other color code and related information are described in "Methods" section. (A) Motif 1 (P Block): the completely conserved proline residue in all DGATs, (B) Motif 2 (FG Block): the completely conserved tryptophan residue followed by a highly conserved glycine residue in all DGATs, (C) Motif 3 (P-1 Block): the almost completely conserved proline residue except one in all DGATs.

The sequences between DGAT1s and DGAT2s are very divergent. No common features between these two subfamilies were reported previously using 7 DGAT1s and 8 DGAT2s from 10 organisms [13]. However, the biochemical function of the two DGAT isoforms is essentially identical in enzymatic assays, suggesting that certain common sequence conservations are probably present between them. Multiple sequence alignment was performed to analyze all 117 DGATs including 59 DGAT1s from 48 organisms and 58 DGAT2s from 44 organisms. Sequence alignment has identified only two completely conserved amino acid residues among all DGATs. One perfectly conserved proline residue corresponds to P248 in AaDGAT1-XP_001658299 and P151 in VfDGAT2-DQ356682.1 (Figure 2A); the other residue, a perfectly conserved phenylalanine residue corresponds to F344 in AaDGAT1-XP_001658299 and F225 in VfDGAT2-DQ356682.1 (Figure 2B). The conserved phenylalanine residue is followed by a glycine residue conserved in all except DGAT1s of *Aedes aegypti, Drosophila melanogaster *and *Tribolium castaneum *(Figure 2B). The other proline residue in Figure 2C is almost completely conserved except DGAT2 of *Ostreococcus tau*ri, which corresponds to P411 in AaDGAT1-XP_001658299 and P276 in VfDGAT2-DQ356682.1. Based on the sequence conservation patterns with these conserved residues as anchors, the conserved sequence motifs among all DGATs are named as Motif 1 (P Block), Motif 2 (FG Block) and Motif 3 (P-1 Block). The highly conserved residues among diverse organisms may be located at the active sites of the enzymes and play important roles in structure, substrate binding and/or catalysis.

### Identification of amino acid residues and sequence motifs conserved in DGAT1s

**Figure 3:**
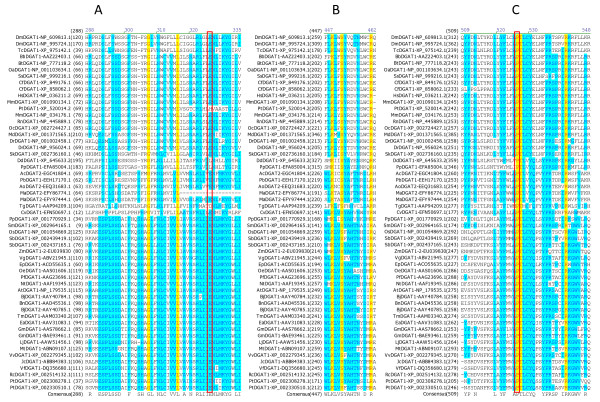
**Identification of completely conserved amino acid residues in sequence motifs 1-3 of DGAT1s**. (A) DGAT1-Motif 1 (GL Block; The boxed E residue is mutated in TmDGAT1), (B) DGAT1-Motif 2 (KSR Block), (C) DGAT1-Motif 3 (PTR Block; The boxed P residues are also conserved in DGAT2s). Multiple sequence alignment was performed using 55 full-length DGAT1 protein sequences from 45 organisms (listed in Table 1). The completely conserved amino acid residues are highlighted in red on yellow. Other color code and related information are described briefly in Figure 2 legend and with details in "Methods" section.

**Figure 4:**
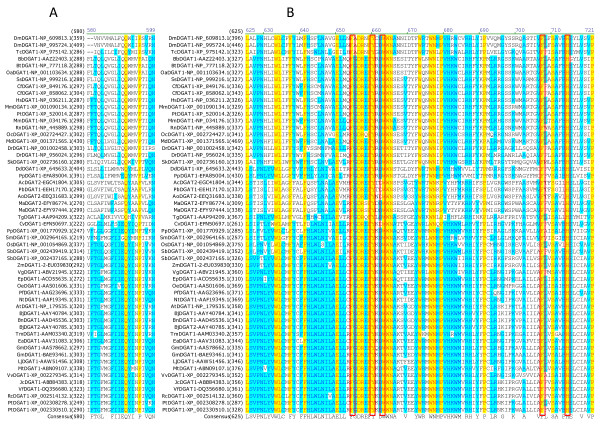
**Identification of completely conserved amino acid residues in sequence motifs 4-5 of DGAT1s**. (A) DGAT1-Motif 4 (QP Block), (B) DGAT1-Motif 5 (LWLFFEFDRFYWWNWWNPPFSHP Block; The first boxed F residues are also conserved in DGAT2s; The boxed Y, W and the second F residues are mutated in TmDGAT1 and the boxed H residue is mutated in MmDGAT1). Refer to Figure 3 legend for additional information.

**Figure 5:**
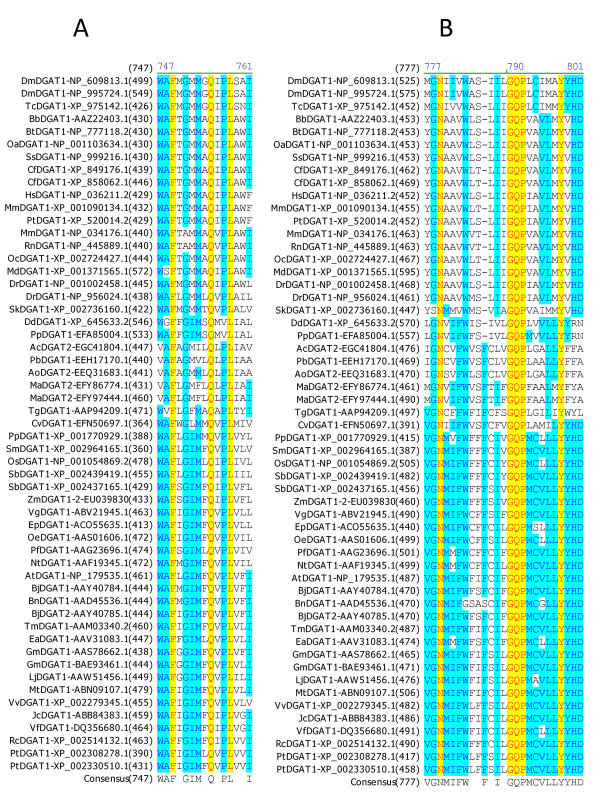
**Identification of completely conserved amino acid residues in sequence motifs 6-7 of DGAT1s**. (A) DGAT1-Motif 6 (FQL Block), (B) DGAT1-Motif 7 (NGQPY Block). Refer to Figure 3 legend for additional information.

Multiple sequence alignment was performed to identify conserved amino acid residues and sequence motifs within the DGAT1 subfamily. Among the 55 full-length DGAT1s, 41 amino acid residues are completely conserved, which correspond to 8.0% of the total 515 residues of DGAT1s. Table 3 shows the positions of the 41 completely conserved residues in DGAT1s from representatives of animal group (mouse), plant group (tung tree) and fungus group (*Dictyostelium discoideum*). These completely conserved residues are located in seven sequence motifs of DGAT1s. Based on the sequence conservation patterns with the completely conserved residues as anchors, the conserved sequence motifs of DGAT1s are named as Motif 1 (GL Block), Motif 2 (KSR Block), Motif 3 (PTR Block) (Figure 3A-C), Motif 4 (QP Block), Motif 5 (LWLFFEFDRFYWWNWWNPPFSHP Block) (Figure 4A-B), Motif 6 (FQL Block) and Motif 7 (NGQPY Block) (Figure 5A-B). The great majority of the conserved residues are located within the carboxyl terminal halves of DGAT1s. Among the completely conserved residues, 33 of them are located within the last 200 residues from the carboxyl termini in Motifs 4-7 (Figures 4, 5), and 23 of them are concentrated in the most conserved region with approximately 100 residues in Motif 6 (Figure 4B). The first two conserved residues (G, L) in Motif 1 start approximately 100 residues from the amino termini (Figure 3A). The next three conserved residues (K, S, R) in Motif 2 start until approximately 200 residues from the amino termini (Figure 3B). The last conserved residue (Y) in Motif 7 ends within the last 20 residues from the carboxyl termini of DGAT1s (Figure 5B).

**Table 3:** The completely conserved residues in DGATs using examples of mouse, tung tree and *Dictyostelium discoideum*.

Organism	DGAT1	DGAT2
**Mouse** **(animal)**	1 MGDRGGAGSS RRRRTGSRVS VQGGSGPKVE EDEVRDAAVS PDLGAGGDAP 51 APAPAPAHTR DKDGRTSVGD GYWDLRCHRL QDSLFSSDSG FSNYR**G**ILNW 101 CVVM**L**ILSNA RLFLENLIKY GILVDPIQVV SLFLKDPYSW PAPCVIIASN 151 IFVVAAFQIE KRLAVGALTE QMGLLLHVVN LATIICFPAA VALLVESITP 201 VGSVFALASY SIMFL**K**LY**S**Y RDVNLWC**R**QR RVKAKAVSTG KKVSGAAAQQ 251 AVSYPDNLTY RDLYYFIFA**P ****T**LCYELNFPR SPRI**R**KRFLL RRVLEMLFFT 301 QLQVGLI**Q**QW MV**P**TIQNSMK PFKDMDYSRI IERLLK**L**AVP NHLI**WL**IF**F**Y 351 WF**F**HSCLNAV A**E**LLQ**F**G**DR**E **FY**RD**WWN**AES VTYF**W**QN**WN**I **P**VHKWCIRHF 401 YK**P**MLRHGSS KWVARTGV**F**L T**S**AFF**H**EYLV SV**P**LRMFRLW A**F**TAMMA**Q**VP 451 **L**AWIVGRFFQ GNYG**N**AAVWV TLII**GQP**VAV LM**Y**VHDYYVL NYDAPVGV 1) G96, 2) L105, 3) K216, 4) S219, 5) R228, 6) P270, 7) T271, 8) R285, 9) Q308, 10) P313, 11) L337, 12) W345, 13) L346, 14) F348, 15) F353, 16) E362, 17) F366, 18) D368, 19) R369, 20) F371, 21) Y372, 22) W375, 23) W376, 24) N377, 25) W385, 26) W388, 27) N389, 28) P391, 29) P403, 30) F419, 31) S422, 32) H426, 33) P433, 34) F442, 35) Q448, 36) L451, 37) N465, 38) G475, 39) Q476, 40) P477, 41) Y483	1 MKTLIAAYSG VLRGERRAEA ARSENKNKGS ALSREGSGRW GTGSSILSAL 51 QDIFSVTWLN RSKVEKQLQV ISVLQWVLSF LVLGVACSVI LMYTFCTDCW 101 LIAVLYFTWL AFDWNTPKKG GRRSQWVRNW AVWRYFRDYF PIQLVKTHNL 151 LTTRNYIFGY H**PH**GIMGLGA FCNFSTEATE VSKKFPGIRP YLATLAGNFR 201 M**P**VL**R**EYLMS GGICPVNRDT IDYLLSKNGS GNAIIIVV**GG **AA**E**SLSSMPG 251 KNAVTLKN**R**K **GF**VKL**A**LRHG ADL**VP**TYS**FG **ENEVYKQVIF EEGSWGRWVQ 301 KKFQKYIGFA PCIFHGRGLF SSDTWGLVPY SKPITTVV**G**E PITVPKLEHP 351 TQKDIDLYHA MYMEALVKLF DNHKTKFGLP ETEVLEVN 1) P162, 2) H163, 3) P202, 4) R205, 5) G239, 6) G240, 7) E243, 8) R259, 9) G261, 10) F262, 11) A266, 12) V274, 13) P275, 14) F279, 15) G280, 16) E339

**Tung tree** **(plant)**	1 MTILETPDNS TDATTSGGAE SSSDLNLSLR RRRTASNSDG AVAELASKID 51 ELESDAGGGQ VIKDPGAEMD SGTLKSNGKD CGTVKDRIEN RENRGGSDVK 101 FTYRPSVPAH RALKESPLSS DNIFKQSHA**G **LFNLCIVV**L**V AVNSRLIIEN 151 IMKYGWLIKT GFWFSSRSLR DWPLLMCCLT LPIFSLAAYL VEKLACRKYI 201 SAPTVVFLHI LFSSTAVLYP VSVILSCESA VLSGVALMLF ACIVWL**K**LV**S** 251 YAHTNFDM**R**A IANSVDKGDA LSNASSAESS HDVSFKSLVY FMVA**PT**LCYQ 301 PSYPRTASI**R **KGWVVRQFVK LIIFTGFMGF IIE**Q**YIN**P**IV QNSQHPLKGD 351 LLYAIERVLK **L**SVPNLYV**WL **CM**F**YCF**F**HLW LNILA**E**LLR**F **G**DR**E**FY**KD**WW** 401 **N**ARTVEEY**W**R M**WN**M**P**VHKWM VRHIYF**P**CLR HKIPRGVALL IT**F**FV**S**AVF**H** 451 ELCIAV**P**CHI FKLWA**F**IGIM F**Q**IP**L**VGITN YLQNKFRSSM VG**N**MIFWFIF 501 CIL**GQP**MCLL L**Y**YHDLMNRK GTTESR 1) G130, 2) L139, 3) K247, 4) S250, 5) R259, 6) P295, 7) T296, 8) R310, 9) Q334, 10) P338, 11) L361, 12) W369, 13) L370, 14) F373, 15) F377, 16) E386, 17) F390, 18) D392, 19) R393, 20) F395, 21) Y396, 22) W399, 23) W400, 24) N401, 25) W409, 26) W412, 27) N413, 28) P415, 29) P427, 30) F443, 31) S446, 32) H450, 33) P457, 34) F466, 35) Q472, 36) L475, 37) N493, 38) G504, 39) Q505, 40) P506, 41) Y512	1 MGMVEVKNEE EVTIFKSGEI YPTNIFQSVL ALAIWLGSFH FILFLVSSSI 51 FLPFSKFLLV IGLLLFFMVI PINDRSKLGQ CLFSYISRHV CSYFPITLHV 101 EDINAFRSDR AYVFGYE**PH**S VFPIGVMILS LGLIPLPNIK FLASSAVFYT 151 **P**FL**R**HIWSWC GLTPATRKNF VSLLSSGYSC ILVP**GG**VQ**E**T FYMKQDSEIA 201 FLKA**R**R**GF**IR I**A**MQTGTPL**V P**VFC**FG**QMHT FKWWKPDGEL FMKIARAIKF 251 TPTIFWGVLG TPLPFKNPMH VVV**G**RPIEVK QNPQPTAEEV AEVQREFIAS 301 LKNLFERHKA RVGYSDLKLE IF 1) P118, 2) H119, 3) P151, 4) R154, 5) G185, 6) G186, 7) E189, 8) R205, 9) G207, 10) F208, 11) A212, 12) V220, 13) P221, 14) F225, 15) G226, 16) E274

***Dictyostelium discoideum*** **(fungus)**	1 MEPIPPSNGN KNNSMDKQPQ QPQQPQQQQQ QQQQQRRDQR NSKLNELNET 51 ERVRNRFISH EFHKLDRTKS RIDAPKISFS DSESESDSEF FLAKRNTNNN 101 NQNNTSPTFS SANGKQSNLT QRKINTQIQS KQPTNNNVQP LTDDEGTINH 151 SNHHHHHHNQ NNNGNNNNNN NNNNNNNKIS TPPKQEEKMT MNGLFTLRPS 201 ILSSESNGSS YR**G**FLNLLLI L**L**ITASFRLV ILNHLLYGIR INLDLYKISE 251 YHRWPGVMIS LMINLFIIAA YLIEKAAAKQ LLPDRICYLL RIINCAAVII 301 VPSGSIIAFS PNPASGIIVM ILICTFSM**K**I I**S**YAYENSKQ **R**KLNPDNKKF 351 VIDPTNTSIY PNNLSLRSTY WFMLV**PT**LVY QLSYPRSPKI **R**KGYLLRRIV 401 EALSLSLLIL WMVN**Q**YML**P**L VQNSIEPLEK IDIVLIVERI MK**L**SLPNLYV 451 **WL**LG**F**YVF**F**H LYLNIVA**E**IT R**F**G**DR**E**FY**RD **WWN**STGLDYF **W**RT**WN**M**P**VHH 501 WMVVLIYT**P**M RRRGFSKNMG YFMC**F**FV**S**AI F**H**ELVISI**P**F HSLKLWG**F**FG 551 IMS**Q**MV**L**IAL TKNLMNGRNL G**N**VIFWISIV L**GQP**LVVLL**Y **YRNFVLENPE 601 WYRNVEPPTS PPVMPFY 1) G213, 2) L222, 3) K329, 4) S332, 5) R341, 6) P376, 7) T377, 8) R391, 9) Q415, 10) P419, 11) L443, 12) W451, 13) L452, 14) F455, 15) F459, 16) E468, 17) F472, 18) D474, 19) R3475, 20) F477, 21) Y3478, 22) W481, 23) W482, 24) N483, 25) W491, 26) W494, 27) N495, 28) P497, 29) P509, 30) F525, 31) S528, 32) H532, 33) P539, 34) F548, 35) Q554, 36) L557, 37) N572, 38) G582, 39) Q583, 40) P584, 41) Y590	1 MVRFVPWNVP LYRRLETMAV AIYAMVLPVC LIMAFNLIVI PLFWGIAIPY 51 LVWMFYFDTK HESGGRRVSL VRNSILWRYF RDYFPISLII NSNYDPKKNY 101 IFAYH**PH**GII SIGAFCNFAT NANNIDEKLP GLKVHLLTLE SNFKI**P**FL**R**D 151 VLMSFGMSSV SKKSCENILN SGAGESICLV V**GG**AE**E**SLDA RPGLNEITLK 201 K**R**K**GF**IKL**A**L VNGASL**VP**VY S**FG**ENDIYDQ VPNPRGSLVR KIQTKIKDLT 251 GIAPPLFMGR GIFNYDFGLL PVRHKIVTVV **G**EPIDIPKIK SPTDQVIEHY 301 HQIYVEALQN LFDKHKNSCA DKETGNLKIN 1) P106, 2) H107, 3) P146, 4) R149, 5) G182, 6) G183, 7) E186, 8) R202, 9) G204, 10) F205, 11) A209, 12) V217, 13) P218, 14) F222, 15) G223, 16) E281

The completely conserved residues in DGAT1s and DGAT2s are underlined and listed below the sequences. The underlined P and F residues are conserved in all DGATs.

### Identification of amino acid residues and sequence motifs conserved in DGAT2s

**Figure 6:**
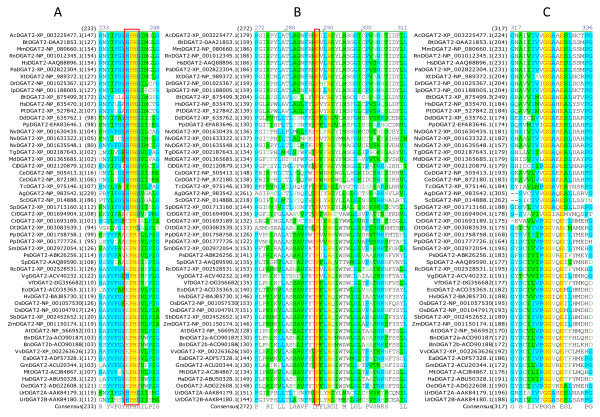
**Identification of completely conserved amino acid residues in sequence motifs 1-3 of DGAT2s**. (A) DGAT2-Motif 1 (PH Block; The boxed H, P and H residues are mutated in MmDGAT2 and ScDGAT2), (B) DGAT2-Motif 2 (PR Block; The boxed P residues are also conserved in DGAT1s), (C) DGAT2-Motif 3 (GGE Block). Multiple sequence alignment was performed using 54 full-length DGAT2 protein sequences from 44 organisms (listed in Table 1). The completely conserved amino acid residues are highlighted in red on yellow. Other color code and related information are described briefly in Figure 2 legend and with details in "Methods" section.

**Figure 7:**
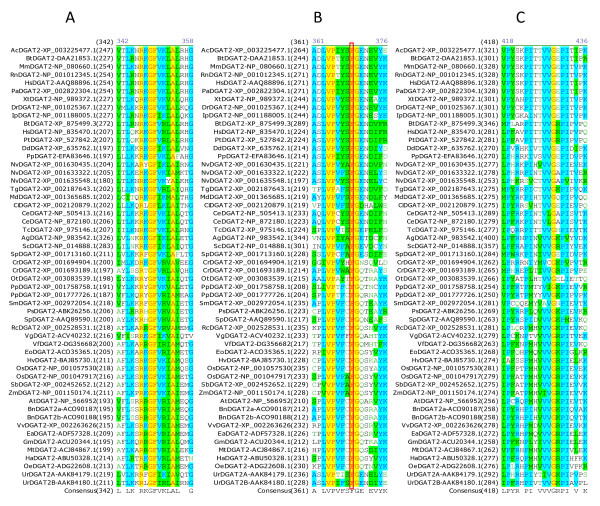
**Identification of completely conserved amino acid residues in sequence motifs 4-6 of DGAT2s**. (A) DGAT2-Motif 4 (RGFA Block), (B) DGAT2-Motif 5 (VPFG Block; The boxed F residues are also conserved in DGAT1s), (C) DGAT2-Motif 6 (G Block). Refer to Figure 6 legend for additional information.

Multiple sequence alignment was also performed using all DGAT2s to identify conserved amino acid residues and sequence motifs within this subfamily. Sixteen residues are completely conserved in the 54 full-length DGAT2s, corresponding to 4.7% of the total 344 residues. Table 3 shows the positions of the 16 completely conserved residues in DGAT2s from representatives of animal group (mouse), plant group (tung tree) and fungus group (*Dictyostelium discoideum*). Based on the sequence conservation patterns with the completely conserved residues as anchors, the conserved sequence motifs of DGAT2s are named as Motif 1 (PH Block), Motif 2 (PR Block), Motif 3 (GGE Block) (Figure 6A-C), Motif 4 (RGFA Block), Motif 5 (VPFG Block) and Motif 6 (G Block) (Figure 7A-C). Similar to DGAT1s, these conserved residues are located at the carboxyl termini of DGAT2s. Eight of them are concentrated within 25 residues in the highly conserved Motifs 4 and 5 of DGAT2s (Figure 7A-B). The first two conserved residues (PH) in Motif 1 start until approximately 100 residues from the amino termini but the last residue (G) ends within the last 50 residues from the carboxyl termini of DGAT1s (Figure 6A and 7C).

### Sequence analysis of important motifs in less conservative regions of DGATs

**Figure 8:**
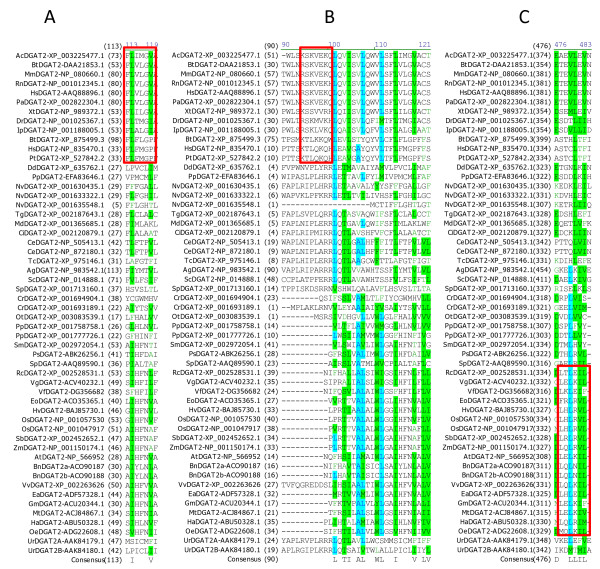
**Sequence analysis of important motifs in less conservative regions of DGAT2s**. Multiple sequence alignment was performed using 54 full-length DGAT2 protein sequences from 44 organisms (listed in Table 1). Color code and related information are described briefly in Figure 2 legend and with details in "Methods" section. The motifs are boxed within the sequence alignment. (A) Putative neutral lipid-binding domain (FLXLXXX in mouse DGAT2) (B) Mitochondrial targeting signal (RXKXXK in mouse DGAT2), (C) ER retrieval motif (LKLEI in tung DGAT2).

Several studies have reported functional motifs in DGATs. However, the conserved sequence motifs identified by our extensive sequence analysis as discussed above do not contain any of the reported putative neutral lipid-binding domain [34], mitochondrial targeting signal [35] or ER retrieval motif [13]. It was reported that mouse DGAT2 contains a consensus sequence (FLXLXXX^n^) for a putative neutral lipid-binding domain [34] which was shown to be present in proteins that either bind to or metabolize neutral lipids [36]. However, this putative motif is only modestly conserved in animal DGAT2s and not present in any plant DGAT2 (Figure 8A). Mouse DGAT2 was also reported to contain a putative mitochondrial targeting signal with positively charged residues (RXKXXK) targeting proteins to mitochondria [35]. This motif is only found in a few animal DGATs but not conserved in any of the plant or fungi DGAT2s (Figure 8B). DGATs are ER-localized enzymes with an ER retrieval motif (LKLEI) at the extreme carboxyl terminus of tung DGAT2 [13]. This sequence analysis shows that this pentapeptide ER-retrieval motif is only modestly conserved in plant DGAT2s but not in animal or fungal DGAT2s, although animal and fugal DGATs are also located to ER (Figure 8C).

### Sequence analysis of important amino acid residues in less conservative regions of DGATs

**Figure 9:**
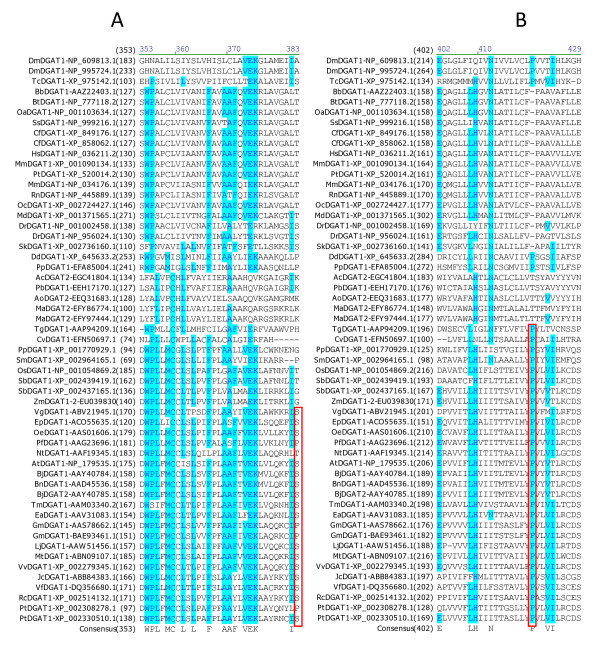
**Sequence analysis of important amino acid residues in less conservative regions of DGAT1s**. (A) The boxed S residue is mutated in *Tropaeolum majus *DGAT1 corresponding to S197, (B) The boxed P residue is mutated in *Tropaeolum majus *DGAT1 corresponding to P216. Multiple sequence alignment was performed using 55 full-length DGAT1 protein sequences from 45 organisms (listed in Table 1). Color code and related information are described briefly in Figure 2 legend and with details in "Methods" section. The amino acid residues studied by mutagenesis and the corresponding conserved residues in other organisms are boxed within the sequence alignment.

The importance of some less conserved residues of DGATs has been demonstrated by site-directed mutants (Table 4). Mutagenesis of a putative SnRK1 target site S197 in *Tropaeolum majus *DGAT1 results in a 38%-80% increase in DGAT1 activity, and over-expression of the mutated TmDGAT1 in *Arabidopsis *results in a 20%-50% increase in oil content on a per seed basis (Table 4) [25]. Figure 9A shows that this serine residue is conserved in most of the plants and some animals except that the same position in DGAT1s alignment is replaced with proline, glycine, threonine and lysine residue in DGAT1s from other organisms. A similar serine residue is not found in any of DGAT2s. Mutagenesis at P216 in *Tropaeolum majus *DGAT1 eliminates almost all of the activity (Table 4) [25]. The P216 residue is completely conserved in plant DGAT1s but is missing in mammalian DGAT1s (Figure 9B).

**Table 4:** Site-directed and natural mutants of DGATs and their effects on enzymatic activity and TAG accumulation.

DGAT	Amino acid sequence with altered residues underlined	Mutation	Activity	Reference
**MmDGAT1-NP_034176.1** **[*Mus musculus*]**	1 MGDRGGAGSS RRRRTGSRVS VQGGSGPKVE EDEVRDAAVS PDLGAGGDAP APAPAPAHTR DKDGRTSVGD 71 GYWDLRCHRL QDSLFSSDSG FSNYRGILNW CVVMLILSNA RLFLENLIKY GILVDPIQVV SLFLKDPYSW 141 PAPCVIIASN IFVVAAFQIE KRLAVGALTE QMGLLLHVVN LATIICFPAA VALLVESITP VGSVFALASY 211 SIMFLKLYSY RDVNLWCRQR RVKAKAVSTG KKVSGAAAQQ AVSYPDNLTY RDLYYFIFAP TLCYELNFPR 281 SPRIRKRFLL RRVLEMLFFT QLQVGLIQQW MVPTIQNSMK PFKDMDYSRI IERLLKLAVP NHLIWLIFFY 351 WFFHSCLNAV AELLQFGDRE FYRDWWNAES VTYFWQNWNI PVHKWCIRHF YKPMLRHGSS KWVARTGVFL 421 TSAFF**H**EYLV SVPLRMFRLW AFTAMMAQVP LAWIVGRFFQ GNYGNAAVWV TLIIGQPVAV LMYVHDYYVL 491 NYDAPVGV	H426A	-100%	[45]

**TmDGAT1-AAM03340.2** **[*Tropaeolum majus*]**	1 MAVAESSQNT TTMSGHGDSD LNNFRRRKPS SSVIEPSSSG FTSTNGVPAT GHVAENRDQD RVGAMENATG 71 SVNLIGNGGG VVIGNEEKQV GETDIRFTYR PSFPAHRRVR ESPLSSDAIF KQSHAGLFNL CIVVLIAVNS 141 RLII**E**NLMKY GWLIDTGFWF SSRSLGDWSI FMCCLTLPIF PLAAFIVEKL VQRNHI**S**ELV AVLLHVIVST 211 AAVLY**P**VIVI LTCDSVYMSG VVLMLFGCIM WLKLVSYAHT SSDIRTLAKS GYKGDAHPNS TIVSCSYDVS 281 LKSLAYFMVA PTLCYQPSYP RSSCIRKGWV VRQFVKLIVF IGLMGFIIEQ YINPIVRNSK HPLKGDFLYA 351 IERVLKLSVP NLYVWLCMFY SFFHLWLNIL AELLRFGDRE F**Y**KD**W**WNAKT VAEYWKMWNM PVHRWMVRHL 421 YFPCLRNGIP KEGAIIIA**F**L VSGAFHELCI AVPCHVFKLW AFIGIMFQVP LVLITNYLQE KFSNSMVGNM 491 IFWFIFCILG QPMCVLLYYH DLINLKEK	E145V S197A P216R Y392A Y392A/W395G F439R	-43% +38-80% -100% -80% -100% -100%	[25]

**MmDGAT2-NP_080660.1** **[*Mus musculus*]**	1 MKTLIAAYSG VLRGERRAEA ARSENKNKGS ALSREGSGRW GTGSSILSAL QDIFSVTWLN RSKVEKQLQV 71 ISVLQWVLS**F ****L**V**L**GVACSVI LMYTFCTDCW LIAVLYFTWL AFDWNTPKKG GRRSQWVRNW AVWRYFRDYF 141 PIQLVKTHNL LTTRNYIFGY **HPH**GIMGLGA FCNFSTEATE VSKKFPGIRP YLATLAGNFR MPVLREYLMS 211 GGICPVNRDT IDYLLSKNGS GNAIIIVVGG AAESLSSMPG KNAVTLKNRK GFVKLALRHG ADLVPTYSFG 281 ENEVYKQVIF EEGSWGRWVQ KKFQKYIGFA PCIFHGRGLF SSDTWGLVPY SKPITTVVGE PITVPKLEHP 351 TQKDIDLYHA MYMEALVKLF DNHKTKFGLP ETEVLEVN	F80A L81A L83A H161A P162G H163A H161A/P162G/H163A	-66% -85% -100% ~50-60% ~50-60% < 20% < 20%	[34]

**ScDGAT2 (Dga1p)-NP_014888.1** **[*Saccharomyces cerevisiae*]**	1 MSGTFNDIRR RKKEEGSPTA GITERHENKS LSSIDKREQT LKPQLES**CC**P LATPFERRLQ TLAVAWHTSS 71 **F**V**L**FSIFTLF AISTPALWVL AIPYMIYFFF DRSPATGEVV NRYSLRFRSL PIWKWY**C**D**YF P**ISLIKTVNL 141 KPTFTLSKNK RVNEKNYKIR LWPTKYSINL KSNSTIDYRN QE**C**TGPTYLF GY**H**P**H**GIGAL GAFGAFATEG 211 **C**NYSKIFPGI PISLMTLVTQ FHIPLYRDYL LALGISSVSR KNALRTLSKN QSI**C**IVVGGA RESLLSSTNG 281 TQLILNKRKG FIKLAIQTGN INLVPVFAFG EVD**C**YNVLST KKDSVLGKMQ LWFKENFGFT IPIFYARGLF 351 NYDFGLLPFR APINVVVGRP IYVEKKITNP PDDVVNHFHD LYIAELKRLY YENREKYGVP DAELKIVG	CC48/49AA C127A C183A C211S C264A C314A F71A L73A Y129A/F130A/P131A H193A H195A	-30% -40% -10% -10% -20% -15% -60% -40% -100% -100% -100%	[33,37]

**ZmDGAT1-2-EU039830** **[*Zea mays*]**	1 MAPPPSMPAA SDRAGPGRDA GDSSSLRLRR APSADAGDLA GDSSGGLREN GEPQSPTNPP PQEQQQHEML 71 YYRASAPAHR RVKESPLSSD AIFRQSHAGL LNLCIVVLIA VNSRLIIENL MKYGLLIRAG FWFSARSLGD 141 WPLLMCCLTL PVFPLVALMA EKLITRKLIG EHVVILLHII ITTSAIVYPV VVTLKCDSAV LSGFVLMFLA 211 SIMWMKLVSY AHTNYDIRVL SKSTEKGAAY GNYVDPENMK DPTFKSLVYF MLAPTLCYQP TYPQTTCIRK 281 GWVTQQLIKC VVFTGLMGFI IEQYINPIVK NSKHPLKGNF LNAIERVLKL SVPTLYVWLC MFYCFFHLWL 351 NIVAELLCFG DREFYKDWWN AKTVEEYWRM WNMPVHKWII RHIYFPCIRK GFSRGVAILI SFLVSAVFHE 421 ICIAVPCHIF KFWAFSGIMF QIPLVFLTRY LHATFKHVMV GNMIFWFF**F**S IVGQPMCVLL YYHDVMNRQA 491 QASR	F469 insertion	+41-107% oil contents	[38]

**BtDGAT1- AAL49962.1** **[*Bos taurus*]**	1 MGDRGGAGGS RRRRTGSRPS IQGGSGPAAA EEEVRDVGAG GDAPVRDTDK DGDVDVGSGH WDLRCHRLQD 71 SLFSSDSGFS NYRGILNWCV VMLILSNARL FLENLIKYGI LVDPIQVVSL FLKDPYSWPA LCLVIVANIF 141 AVAAFQVEKR LAVGALTEQA GLLLHGVNLA TILCFPAAVA FLLESITPVG SVLALMVYTI LFLKLFSYRD 211 VNLWCRERRA GAKAKAALAG K**K**ANGGAAQR TVSYPDNLTY RDLYYFLFAP TLCYELNFPR SPRIRKRFLL 281 RRLLEMLFLT QLQVGLIQQW MVPAIQNSMK PFKDMDYSRI VERLLKLAVP NHLIWLIFFY WLFHSCLNAV 351 AELMQFGDRE FYRDWWNSES ITYFWQNWNI PVHKWCIRHF YKPMLRRGSS KWAARTAVFL ASAFFHEYLV 421 SIPLRMFRLW AFTGMMAQIP LAWIVGRFFR GNYGNAAVWL SLIIGQPVAV LMYVHDYYVL NREAPAAGT	K232A	Devoid of milk secretion	[41]

**Figure 10:**
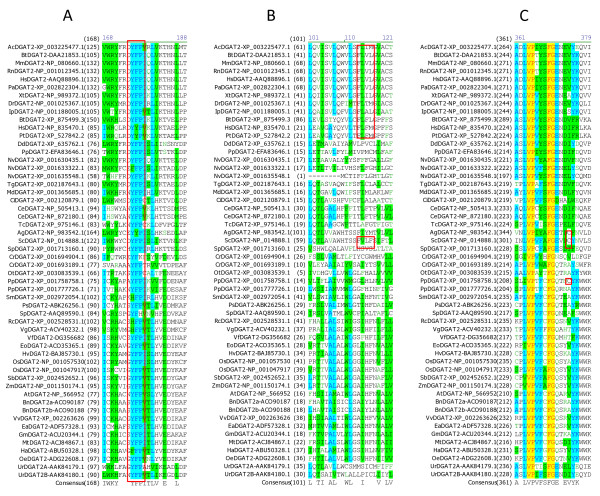
**Sequence analysis of important amino acid residues in less conservative regions of DGAT2s**. (A) YFP motif (The boxed Y, F and P residues are mutated in baker's yeast DGAT2 corresponding to Y129/F130/P131), (B) The boxed F, L and L residues are mutated in mouse DGAT2 corresponding to F80, L81 and L83 and in baker's yeast DGAT2 corresponding to F71 and L73, (C) The boxed C residue is mutated in baker's yeast DGAT2 corresponding to C314. Multiple sequence alignment was performed using 54 full-length DGAT2 protein sequences from 44 organisms (listed in Table 1). Color code and related information are described briefly in Figure 2 legend and with details in "Methods" section. The amino acid residues studied by mutagenesis and the corresponding conserved residues in other organisms are boxed within the sequence alignment.

A highly conserved region with a consensus sequence of "YFP" in DGAT2s is essential for enzymatic activity of DGAT2 from *Saccharomyces cerevisiae *(Table 4) [33]. These three residues are highly conserved and located before Motif 1 but none of the three amino acid residues is completely conserved among all DGAT2s in our analysis using 54 full-length DGAT2s (Figure 10A). The consensus "YFP" is replaced with "YYP" in DGAT2s of human, chimpanzee (*Pan troglodytes*) and *Ashbya gossypii*, "FFP" in *Helianthus annuus *and *Nematostella vectensis*, and "HFP" in caster bean (*Ricinus communis*), *Vernonia galamensisand*, and *Selaginella moellendorffii*. It was reported that a unique region is present in DGAT2 of *Saccharomyces cerevisiae *[33], but in our expanded analysis, a similar region is also found in *Ashbya gossypii *(data not shown).

Mutations at F80/L81/L83 in mouse DGAT2 [34] and F71/L73 in baker's yeast DGAT2 [33] result in partial loss of the activity (Table 4). This region is only relatively conserved in some animal DGAT2s (Figure 10B). Finally, ScDGAT2 has a unique cysteine residue (C314) which is not involved in catalysis but may be located near the active site or related to proper folding of the protein [37]. However, this residue is only found in DGAT2s from baker's yeast and the other fungi *Ashbya gossypii *and *Physcomitrella patens*, but is not present in the same position of the alignment in any of the other 51 DGAT2s or any of the 55 DGAT1s analyzed (Figure 10C).

### Sequence analysis of important amino acid residues of DGATs shown in natural mutants

**Figure 11:**
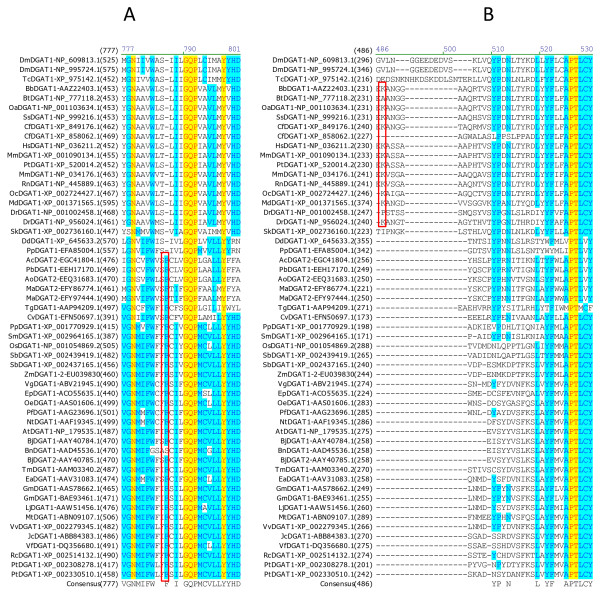
**Sequence analysis of important amino acid residues of DGAT1s shown in natural mutants**. Multiple sequence alignment was performed using 55 full-length DGAT1 protein sequences from 45 organisms (listed in Table 1). The completely conserved amino acid residues are highlighted in red on yellow. Other color code and related information are described briefly in Figure 2 legend and with details in "Methods" section. The amino acid residues affected by natural mutation and the corresponding conserved residues in other organisms are boxed within the sequence alignment. (A) maize DGAT1-2 F468, (B) cattle DGAT1 K232A.

The importance of some relatively conserved residues in TAG biosynthesis has been demonstrated by two well-known natural mutants in corn and cattle. A phenylalanine insertion (F469) in DGAT1-2 increases oil and oleic-acid contents in maize. Ectopic expression of the high-oil DGAT1-2 allele increases oil and oleic-acid contents by up to 41% and 107%, respectively (Table 4) [38]. This phenylalanine residue is conserved in all plants except *Brassica napus *[GenBank:AAD45536.1] and conserved in all fungi except mold (*Dictyostelium discoideum *and *Polysphondylium pallidum*) (Figure 11A). Rape DGAT1 is the only sequence in the plant group with a serine residue in the place of phenylalanine in the sequence alignment (Figure 11A). Since this gene is isolated from suspension cultures of *Brassica napus *[39] and the native form is not available, it is not known if this replacement was caused by mutations due to cell culture conditions, considering that cell culture could cause significant changes in gene expression [40]. It is interesting to note that a similar phenylalanine residue is not present in any of the animal DGAT1s or any of DGAT2s.

In cattle, a nonconservative substitution of lysine by alanine (K232A) in DGAT1 (Table 4) is directly responsible for the quantitative trait loci (QTL) variation with the lysine-encoding allele being associated with higher milk fat content [41]. Figure 11B shows that the lysine residue is conserved in mammalian DGAT1s but not in plants, or fungi, or other animals (fly, frog, insect and worm) except one of the two forms from dog and zebrafish. The wild-type cattle DGAT1 shows the normal lysine at 232 position [GenBank:AAL49962.1] (Table 4). A similar lysine residue is not found in any of DGAT2s.

## Discussion

### DGAT classification

The nomenclature of proteins derived from DNA sequences in GenBank databases can lead to confusion in some cases. One of the utilities of this extensive sequence analysis is to use the completely conserved amino acid residues in respective sequence blocks of DGAT1 and DGAT2 subfamilies as signatures of DGAT proteins for classification. It is generally accepted that DGATs are divided into DGAT1 and DGAT2 subfamilies. However, more than two forms of DGATs are present in a number of species (Table 1). Phylogenetic analysis and multiple sequence alignment classify all 117 DGAT protein sequences into DGAT1 and DGAT2 subfamilies (Figures 1, 3, 4, 5, 6, 7). Furthermore, six DGATs currently designated as DGAT2s in the databases are reclassified here as DGAT1s due to their close alignment with DGAT1s and these sequences containing all 41 completely conserved amino acid residues found in DGAT1s. These six reclassified sequences are [GenBank:EGC41804.1, GenBank:EEQ31683.1, GenBank:AAY40785.1, GenBank:AAD40881.1, GenBank:EFY86774.1 and GenBank:EFY97444.1] (Table 1).

DGATs which diverge from the DGAT1 and DGAT2 subfamilies have been recently reported from *Arabidopsis thaliana *[GenBank:AAN31909.1] [16], Burning Bush (*Euonymus alatus*) [GenBank:GU594061.1] [14], peanut (*Arachis hypogaea*), [GenBank:AY875644.1] [15], caster bean (*Ricinus communis*) [GenBank:XP_002519339.1] and yeast (*Rhodotorula glutinis*) [GenBank:DG315417.1]. Phylogenetic analysis and multiple sequence alignment indicate that these sequences are completely different from DGAT1s and DGAT2s. In fact, none of the completely conserved residues in DGAT1s (41 residues) and DGAT2s (16 residues) is present in these new DGATs in the multiple sequences alignment (data not shown). This sequence divergence is in contrast to the general belief that the active sites of enzymes should be conserved during the evolution because all catalyze the same/similar biochemical reaction. Therefore, this sequence divergence raises an important question how completely different proteins could perform the same biochemical reaction.

### DGAT properties and amino acid composition

This study has analyzed the properties and amino acid composition of 109 full-length DGATs from 70 organisms. The average DGAT1s are 171 amino acid residues longer than DGAT2s resulting in approximately 20 kDa difference in the molecular mass. Other DGAT properties are similar: both are basic proteins under neutral pH with high isoelectric points (Table 2). The frequency of functional amino acid residue groups between DGAT1 and DGAT2 subfamilies is also very similar in terms of charged residues, acidic residues, basic residues, polar residues and hydrophobic residues (Table 2). The remarkable feature of DGAT1s and DGAT2s is that both subfamilies of proteins contain more than 40% of hydrophobic residues (Table 2). These high amounts of hydrophobic residues in DGATs are in agreement with them being integral membrane proteins [33,34] with multiple transmembrane domains [13,33,34], localized to endoplasmic reticulum of plant and animal cells [13,34], and associated with mitochondria in COS-7 cells [35,42] and lipid bodies in 3T3-L1 adipocytes [42]. The membrane association of the proteins presents extra huddle to purification of recombinant DGATs from any source [43,44].

### Catalytic and regulatory domains of DGATs

Generally speaking, critical amino acid residues of proteins are conserved during the evolution because they are essential for enzymatic activity. The conserved amino acid residues are clustered at the active centers of the enzymes. Multiple sequence alignment has shown that DGAT1s and DGAT2s have 41 and 16 completely conserved amino acid residues, respectively. Most of them are located at the carboxyl termini of DGATs (Table 3). This sequence analysis suggests that the catalytic domains of DGATs are located at the carboxyl termini of the proteins. This is supported by mutations of some completely conserved amino acid residues in the C-termini of these proteins resulted in complete loss of the enzymatic activity of DGATs (see below). This suggestion is in line with our previous assignment of the catalytic domains of ADPGlc-dependent α-1,4-glucosyltransferases and α-1,6-glucan hydrolases from plants and prokaryotes at the carboxyl termini of the enzymes because of the presence of the conserved amino acid residues and sequence motifs in the different isoforms from diverse organisms [6,9].

Several lines of evidence suggest that the regulatory domains of DGATs are located at the amino termini of the proteins. First, a recent study showed that the amino terminal domain of DGAT1 of mouse is not required for the catalytic activity of DGAT1 but may be involved in regulating enzyme activity and dimer/tetramer formation [45]. Second, the N-terminal region of mouse DGAT2 or yeast DGAT2 is not essential for DGAT activity *in vitro *[33,35]. Finally, mutagenesis of a putative protein kinase SnRK1 (SNF1-related kinase 1) target site at S197 to alanine in TmDGAT1 results in a 38%-80% increase in DGAT1 activity, and over-expression of the mutated TmDGAT1 in *Arabidopsis *results in a 20%-50% increase in oil content on a per seed basis [25]. This serine residue is conserved in most of the plants and located at the N-termini of DGAT1s (Figure 9A). All of the above mentioned sequence analysis and experimental evidence support the concept that the catalytic and regulatory domains of DGATs are located at the C- and N-termini of the enzymes, respectively.

### Functional significance of less conserved motifs in DGAT2s

Recent studies have reported functional motifs in DGATs including putative neutral lipid-binding domain (FLXLXXX^n ^in mouse DGAT2) [34], mitochondrial targeting signal (RXKXXK in mouse DGAT2) [35] and ER retrieval motif (LKLEI in tung DGAT2) [13]. However, the conserved sequence motifs identified by our extensive sequence analysis do not contain any of these reported motifs. In our analysis, the putative neutral lipid-binding domain [34] which was shown to be presented in proteins that either bind to or metabolize neutral lipids [36], is only modestly conserved in animal DGAT2s and not present in any plant DGAT2 (Figure 8A). Similarly, the putative mitochondrial targeting signal is only found in a few animal DGAT2s but not conserved in any plant or fungi DGAT2 (Figure 8B). This sequence analysis also shows that the pentapeptide (LKLEI) ER-retrieval motif identified at the extreme carboxyl terminus of tung DGAT2 [13] is only modestly conserved in plant DGAT2s but not in animal or fungus DGAT2s (Figure 8C). All these studies point out that less conserved regions in a subset of DGATs may play specific roles in TAG biosynthesis in that particular subset of organisms.

### Functional significance of the completely conserved residues

Multiple sequence alignment has shown that 55 DGAT1s and 54 DGAT2s have 41 and 16 completely conserved amino acid residues, respectively, although only two residues are completely conserved among all DGATs (Table 3). It is likely that these completely conserved amino acid residues are critical for DGAT enzymatic activities. These residues may be involved in substrate binding, direct catalysis, and/or maintenance of protein structure including oligomer formation. The importance of some conserved residues in DGAT1s has been demonstrated by site-directed mutagenesis (Table 4). Mutagenesis at H426 in mouse DGAT1 to alanine impairs the ability of DGAT1 to synthesize triacylglycerols, retinyl and wax esters in an "*in vitro*" acyltransferase assay [45]. This histidine residue is completely conserved in Motif 5 of all DGAT1s (Figure 4B). Similarly, mutagenesis at Y392, W395 and F439 in *Tropaeolum majus *DGAT1 eliminates nearly all activity [25]. These three residues are also completely conserved in Motif 5 of all DGAT1s (Figure 4B). All four residues are located in the most conserved region of DGAT1s in which 23 completely conserved residues are located in Motif 5 of the multiple sequence alignment (Figure 4B).

The importance of the completely conserved residues in DGAT2s is also supported by site-directed mutagenesis. Mutagenesis at H161, P162 and H163 sites, and the triple mutant in mouse DGAT2 results in a substantial loss of activity (Table 4) [34]. Mutation at the corresponding sites at H193 and H195 in DGAT2 of baker's yeast results in complete loss of the activity (Table 4) [33]. These three resides are located in the highly conserved Motif 1 (PH Block) of DGAT2s (Figure 6A). These results suggest that they may be located at the active center of DGAT1s, but the precise roles of these residues either involved in substrate binding or catalysis are not clear. Further experiments are required to assess the contribution of the other completely conserved residues to the enzymatic activity of DGATs.

### Functional significance of the less-well conserved residues in site-directed mutants

The importance of some less conserved residues in DGATs has also been demonstrated by site-directed mutagenesis (Table 4). As described above, mutation at S197 (a putative SnRK1 target site) in TmDGAT1 results in a 38%-80% increase in DGAT1 activity. This serine residue is conserved in most of the plants (Figure 9A). In addition, mutagenesis at E145 in Motif 1 of *Tropaeolum majus *DGAT1 results in the loss of almost half of the activity [25]. This glutamate residue is conserved in all plant DGAT1s and most other DGAT1s except bird, chimpanzee, *Dictyostelium discoideum*, *Polysphondylium pallidum *and *Metarhizium acridum *(Figure 3A). Mutagenesis at P216 in *Tropaeolum majus *DGAT1 eliminates almost all of the activity [25]. P216 is completely conserved in plant DGAT1s but is missing in all mammalian DGAT1s (Figure 9B). Mutation at Y129/F130/P131 in DGAT2 of baker's yeast results in a complete loss of the activity [33]. These three residues are highly conserved but none of them is completely conserved among all DGAT2s in our analysis using 54 full-length DGATs (Figure 10A). Mutations at F80/L81/L83 in mouse DGAT2 [34] and F71/L73 in baker's yeast DGAT2 [33] result in partial loss of the DGAT activity (Figure 10B). Finally, ScDGAT2 has a unique cysteine residue (C314) which is not involved in catalysis but may be located near the active site or related to proper folding of the protein [37]. However, this residue is only found in DGAT2s from baker's yeast and the other two fungi *Ashbya gossypii *and *Physcomitrella patens*, but is not present in any of the other 51 DGAT2s or any of the 55 DGAT1s analyzed (Figure 10C). Nonetheless, site-directed mutagenesis indicates that these less conserved residues, although not essential, contribute to the full activity of DGATs.

### Functional significance of the relatively conserved residues in natural mutants

Two well-known natural mutants in corn and cattle demonstrate the importance of some relatively conserved residues in TAG biosynthesis (Table 4). Genetic mapping has identified a high-oil QTL (qHO6) that affects maize seed oil and oleic-acid contents associated with DGAT1-2 [38]. A phenylalanine insertion (F469) in DGAT1-2 is responsible for the increased oil and oleic-acid contents. Ectopic expression of the high-oil DGAT1-2 allele increases oil and oleic-acid contents by up to 41% and 107%, respectively [38]. This phenylalanine residue is conserved in all plants except *Brassica napus *(rape, AAD45536.1) and conserved in all fungi except mold (*Dictyostelium discoideum *and *Polysphondylium pallidum*) (Figure 11A). It is not present in any of the animal DGAT1s or any of DGAT2s. This case suggests that oil content can be potentially improved in transgenic plants by introducing site-specific amino acid substitutions/changes in DGATs.

DGAT1 knockout mice are completely devoid of milk secretion, most likely because of deficient triglyceride synthesis in the mammary gland [18]. DGAT1 sequences from pooled DNA show significant frequency shifts at several residue positions between groups of animals with high and low breeding values for milk fat content in different breeds [41]. Substitution of lysine by alanine (K232A) is directly responsible for the QTL variation with the lysine-encoding allele being associated with higher milk fat content [41]. Both DGAT1 alleles are expressed in Sf9 cells, an insect expression system, and characterized the expressed proteins. The K allele, causing an increase in milk fat percentage in the live animal, is characterized by a higher Vmax in producing triglycerides than the A allele [46]. This lysine residue is conserved in mammals but not in plants, fungi or other animals except one of the two forms from dog and zebrafish (Figure 11B). This case also suggests that lipid content can be improved in transgenic animals by bioengineering specific amino acid residues of DGATs.

## Conclusions

Understanding the precise roles of DGATs may help to create transgenic plants with value-added properties and provide information for therapeutic intervention for obesity and related diseases because DGATs catalyze the final and rate-limiting step of TAG biosynthesis in eukaryotic organisms. This report analyzed 117 DGAT sequences from 70 organisms ranging from plants, animals and fungi to aid our understanding of the structure-function relationship of these important enzymes. The report identified conserved sequence motifs and amino acid residues in all 117 DGATs and DGAT1 and DGAT2 subfamilies, reassigned some DGAT subfamily members based on the phylogenetic analysis and sequence similarities, and discussed the importance of some conserved residues with site-directed and natural mutants. One interesting observation is that the newly reported DGAT3 and DGAT4 sequences do not contain any of the completely conserved residues in DGAT1s (41 residues) and DGAT2s (16 residues) in the multiple sequences alignment. This sequence divergence is in contrast to the general belief that the active sites of enzymes should be conserved during the evolution because all catalyze the same/similar biochemical reaction. Therefore, this sequence divergence raises an important question how proteins with completely different amino acid sequences could perform the same biochemical reaction, although some variations of the conserved sequence motifs and amino acid residues are expected when more sequences of DGATs are used in the multiple sequence alignment.

It has been well-documented that many of the enzymes in the oil biosynthesis pathway are not stable. Although the precise reasons are unknown, it is possible that plants develop a feedback mechanism to regulate the optimal amount of enzymes so that the biophysical properties of ER membranes are functionally intact without dramatic alterations by over-expressed enzymes in the host. If this is the case, it may be advantageous to introduce genes with low copy numbers but with high catalytic efficiency. This concept is supported by three studies with plants (S197 in *Arabidopsis *and F469 in corn) and animals (K232 in cattle) which demonstrate the potential to increase oil/fat production by altering a single amino acid residue of DGAT1. Therefore, the sequence analysis should facilitate studying the structure-function relationship of DGATs with the ultimate goal of identifying critical amino acid residues. This will guide the construction of superb enzymes for metabolic engineering and rational design of DGAT inhibitors to be used for obesity and related diseases.

## Methods

### Database search of DGATs

DGAT sequences were obtained from Blastp search [47,48] using tung tree (*Vernicia fordii*) DGAT sequences [GenBank:DQ356680.1] (DGAT1) and [GenBank:DQ356682.1] (DGAT2) [13] against the National Center for Biotechnology Information (NCBI)'s non-redundant protein sequence databases http://blast.ncbi.nlm.nih.gov/Blast.cgi. Additional DGAT sequences were obtained using DGAT1 and DGAT2 search term in NCBI's Protein database. http://www.ncbi.nlm.nih.gov/protein. A total of 109 full-length and 8 near full-length DGATs were obtained from 70 organisms including plants (such as *Arabidopsis*, barley, caster bean, cauliflower, corn, rape, rice, sorghum, soybean, tobacco, tung tree), animals (such as bird, chimpanzee, cow, dog, fish, fly, frog, monkey, mosquito, mouse, pig, rabbit, rat, sheep, worm), fungi (such as yeast, mold, moss) and human. The names of DGATs used in the analysis and their corresponding organisms, classification of DGAT subfamily, and the GenBank accession numbers are presented in Table 1. The name of each protein sequence consists of the initials of the organism followed by the assigned subfamily of DGATs in the databases and the GenBank accession number.

### Protein analysis

The properties and amino acid compositions of DGATs were analyzed using Vector NTI software (Invitrogen) [49]. Statistics was performed using Microsoft Excel.

### Phylogenetic analysis

Phylogenetic analysis was used to study the presumed evolutionary relationships among the 117 DGATs from 70 organisms. This analysis was performed using the Vector NTI software (Invitrogen) based on the Neighbor-Joining method of Saitou and Nei [50]. The numbers in the parenthesis following DGAT names are the calculated distance values which reflect the degree of divergence between all pairs of DGAT sequences analyzed.

### Multiple sequence alignment

Multiple sequence alignment was performed using the ClustalW algorithm [51,52] of the AlignX program of the Vector NTI software. This method is based on algorithms that assign scores to aligned residues and detect sequence similarities. Identical amino acid residues in alignment have higher scores than those not identical and less similar residues. Each DGAT sequence name is on the left of the alignment followed by the position of amino acid residue of DGAT protein sequence in the alignment. The numbers at the top of the alignment are the positions of the multiple sequence alignment. The letters at the bottom of the alignment are the consensus residues. Color codes for amino acid residues are as follows: 1) red on yellow: consensus residue derived from a completely conserved residue at a given position; 2) black on green: consensus residue derived from the occurrence of greater than 50% of a single residue at a given position; 3) blue on cyan: consensus residue derived from a block of similar residues at a given position; 4) green on white: residue weakly similar to consensus residue at a given position; 5) black on white: non-similar residues.

## Lists of abbreviations

DGAT: diacylglycerol acyltransferase; QTL: quantitative trait loci; TAG: triacylglycerol

## Competing interests

The author declares that they have no competing interests.

## Authors' contributions

HC carried out all aspects of the study including literature review, study design, database search, protein analysis, phylogenetic analysis and multiple sequence alignment. HC also wrote the draft, revised the manuscript after internal review, obtained submission permission from USDA-ARS and approved the final manuscript.
